# Unbiased and comprehensive identification of virus-derived circular RNAs in a large range of viral species and families

**DOI:** 10.1371/journal.ppat.1013448

**Published:** 2025-09-11

**Authors:** Alexis S. Chasseur, Maxime Bellefroid, Mathilde Galais, Meijiao Gong, Pierre Lombard, Sarah Mathieu, Amandine Pecquet, Estelle Plant, Camille Ponsard, Laure Vreux, Carlo Yague-Sanz, Benjamin G. Dewals, Nicolas A. Gillet, Benoît Muylkens, Carine M. Van Lint, Damien Coupeau

**Affiliations:** 1 Namur Research Institute for Life Sciences (NARILIS), Integrated Veterinary Research Unit (URVI), University of Namur, Namur, Belgium; 2 Service of Molecular Virology, Department of Molecular Biology (DBM), Université Libre de Bruxelles (ULB), Gosselies, Belgium; 3 Department of Infectious and Parasitic Diseases, Faculty of Veterinary Medicine – FARAH, University of Liège, Liège, Belgium; 4 Namur Research Institute for Life Sciences (NARILIS), Molecular Physiology Research Unit (URPhyM), University of Namur, Namur, Belgium; 5 Walloon Excellence in Life Sciences and Biotechnology (WELBIO) Department, WEL Research Institute, Wavre, Belgium; University of Illinois at Chicago College of Medicine, UNITED STATES OF AMERICA

## Abstract

Non-coding RNAs play a significant role in viral infection cycles, with recent attention focused on circular RNAs (circRNAs) originating from various viral families. Notably, these circRNAs have been associated with oncogenesis and alterations in viral fitness. However, identifying their expression has proven more challenging than initially anticipated due to unique viral characteristics. This challenge has the potential to impede progress in our understanding of viral circRNAs. Key hurdles in working with viral genomes include: (1) the presence of repetitive regions that can lead to misalignment of sequencing reads, and (2) unconventional splicing mechanisms that deviate from conserved eukaryotic patterns. To address these challenges, we developed vCircTrappist, a bioinformatic pipeline tailored to identify backsplicing events and pinpoint loci expressing circRNAs in RNA sequencing data. Applying this pipeline, we obtained novel insights from both new and existing datasets encompassing a range of animal and human pathogens belonging to Herpesviridae, Retroviridae, Adenoviridae, Flaviviridae and Orthomyxoviridae families. Subsequent RT-PCR and Sanger sequencings validated the accuracy of the developed bioinformatic tool for a selection of new candidate virus-derived circRNAs. These findings demonstrate that vCircTrappist is an open and unbiased approach for comprehensive identification of virus-derived circRNAs.

## Author summary

Circular RNAs differ from linear RNAs in that they lack free ends, making them more stable in the extracellular environment. Previous studies have shown that they can play important roles in viral life cycles, including infection and pathogenesis. However, their biogenesis from viral genomes remains poorly understood. In humans, circRNAs are known to be produced through a specific canonical mechanism. In viruses, however, our team and others have described non-canonical circular RNAs.

In this study, we present a novel pipeline that outperforms the current gold standard in the detection of non-canonical circular RNAs. Using both simulated and real infection datasets, we evaluated its performance and confirmed its robustness. This approach enabled us to identify circular RNAs produced by oncogenic viruses and to propose potential functions for these atypical transcripts, which do not follow the classically described biogenesis pathways. While further research will be needed to elucidate their precise roles, this study paves the way for their systematic discovery and a deeper understanding of viral replication and pathogenesis. In the long term, these circular RNAs may serve as valuable targets for the development of new therapeutic strategies against viral diseases.

## Introduction

While not classified as living organisms, viruses show remarkable adaptability to their environment. They exhibit an exceptional ability to co-opt and manipulate various cellular pathways, using the cellular machinery for their regulation, replication and dissemination. Viruses impact every facet of the central dogma of biology, spanning from genomic [1] to proteomic levels [2].

Circular RNAs (circRNAs) have been implicated in diverse molecular mechanisms. Initially considered non-coding, some of them have since been demonstrated to undergo translation, akin to conventional linear mRNAs. In the field of viral biology, circRNAs have been identified across numerous viral families (reviewed in [3–5]). While the majority of their functions remain to be elucidated, it is worth emphasizing that their diversity and multiple splicing patterns involved in their biogenesis render their individual study challenging [6]. Nonetheless, in-depth characterization of select circRNA candidates within specific viral families has provided promising insights into their potential roles in pathogenesis. One notable example is circE7 from Human Papillomavirus (HPV) 16, which has been demonstrated to undergo translation, encoding the oncoprotein E7 through an RNA methylation-mediated mechanism [7]. Furthermore, roles in pathogenesis have been uncovered for herpesviruses [8,9], influenzavirus (IAV) H1N1 [10], and Hepatitis C virus (HCV) [11].

Biogenesis of viral circRNAs presents an intriguing puzzle. While the generation of cellular circRNAs was mostly associated with a canonical backsplicing mechanism involving the U2 splicing machinery, viral circRNAs appear to be partly produced through as-yet-unknown mechanisms. This phenomenon has been observed primarily in RNA viruses [10,12–15], but it is also noteworthy in DNA viruses, exemplified by Marek’s Disease Virus (MDV, also known as Gallid Herpesvirus 2 – GaHV-2) [16], which displays numerous circRNAs with splicing patterns not associated with the canonical GU-AG motif, surrounding upstream and downstream ends of removed introns. It is noteworthy that the GU-AG backsplicing signature, associated with the U2 machinery, is a pattern in circRNA identification by most existing bioinformatics tools. However, this signature is not representative of the diversity of circRNA junction observed in the viral world. For instance, in the case of the Respiratory syncytial virus (RSV) [14], an AU-UA pattern was observed, but a clear association with any splicing mechanism remains elusive. A recent study exploring HCV circRNAs [11] has suggested that the generation of functionally active viral circRNAs might result from an alternative mechanism due to the absence of viral RNA within the nucleus, where canonical splicing occurs. In the case of the coronavirus Murine Hepatitis Virus (MHV), a study from Yang and colleagues has underscored the importance of the viral exonuclease NSP14 in circRNA biogenesis [17].

To decipher the intricacies of the viral infectious cycle and its impact on host cellular processes, it is imperative to comprehensively catalog all viral factors expressed during infection. This identification phase presents technical challenges. Within this context, the study of viral circRNAs is marked by pronounced practical difficulties. Indeed, viral circRNAs might be produced by various mechanisms and viral genomes are characterized by the presence of numerous repeated regions, these two features making circRNA identification more challenging in viruses. While a lot of programs are already available to identify new circRNA candidates (reviewed in [18]), only three are commonly used in the context of viral infections: CIRI2 [19], find_circ [20] and circRNA_finder [21]. Using these, a database was created by Fu and collaborators and named VirusCircBase [22]. However, these currently available tools all use the fixed GU/AG signal to identify circRNAs. Their second limitation is the fact that they do not take into account the peculiarities of viral genomes described above. In the present study, we introduce vCircTrappist, a novel bioinformatics pipeline tailored to the distinctive attributes of viral genomes, thereby surmounting the constraints associated with prevailing circRNA analysis tools. vCircTrappist uses data generated by Illumina high-throughput sequencing to precisely detect atypical splicing events, called backsplice junctions (BSJ), enabling the identification of uncharted circRNA candidates. Stringent filters, designed to exclude potential artifacts stemming from viral features such as genomic repetitive elements, are integrated to curtail false positives. Moreover, vCircTrappist is engineered to discern circRNAs exhibiting non-canonical splicing patterns from the canonical U2 ones. Leveraging this innovative framework, and by using newly and pre-existing datasets, we have successfully uncovered circRNAs expressed by diverse viral families, some of which were experimentally validated. This pioneering program promises to enrich resources like VirusCircBase [22] with a plethora of circRNA candidates, thereby substantially elevating the discovery rate of significant viral circRNAs.

## Materials and methods

### Cell lines and viruses

The Embryonic Stem Cell-Derived Line 1 (ESCDL-1) was kindly given to us by Caroline Denesvre. It was cultured, as indicated in Vautherot *et al* [23], at 37°C – 5% CO2 in Dubelcco’s Modified Eagle Medium (DMEM) F12 (32500035 – Gibco) supplemented in 1% non-essential amino acids (11140050 – Gibco), 1mM sodium pyruvate (11360070 – Gibco), 50U/mL of penicillin and streptomycin (15070063 – Gibco) and 10% fetal bovine serum (FBS) (10270106 – Gibco). They were infected with the very virulent strain of MDV, RB-1B. In this case, the cells were first transfected using a viral GFP bacmid where the internal repeat long is lacking, as described in [24]. One million cells were transfected using the Amaxa fibroblast kit (VPI-1002 – Lonza) and the program F-024, following the manufacturer’s protocol. After that, the infection was propagated 3 times to reach around 80% of infected cells in a culture of three million cells. We removed bioinformatically the internal repeats in the sequence for the subsequent analysis.

The two productive bovine leukemia virus (BLV)-infected ovine cell lines L267_LTaxSN_ [1] and YR2_LTaxSN_ [25] used in this study constitutively express the viral transactivator TaxBLV as a result of transduction with the pLTaxSN retroviral vector of native L267 [26] or YR2 [27,28] cells, respectively. The ovine cell lines L267 and YR2 were established from the L267 B-cell lymphoma and M395 B-cell leukemia, respectively, developed by BLV-infected sheep (accession number: KT122858.1) [29]. All BLV-infected cell lines were maintained in Opti-MEM medium (31985070 – Gibco) supplemented with 10% FBS (10270106 – Gibco), 1 mM sodium pyruvate (11360070 – Gibco), 2 mM L-glutamine (25030149 – Gibco), 1% non-essential amino acids (11140050 – Gibco) and 100 μg/ml kanamycin monosulphate (KAN0025 – Formedium).

The 293T-BLV samples were obtained by calcium-phosphate transfection (CalPhos Mammalian Transfection Kit; 631312 – TaKaRa) of 293T cells (CRL-3216 – ATCC) with the pBLV344 plasmid containing a complete and infectious proviral copy of BLV [29]. Fourty-eight hours post-transfection, RNA was extracted as described below. 293T cells were maintained in DMEM (11965092 – Gibco) supplemented with 10% FBS (10270106 – Gibco), 1 mM sodium pyruvate (11360070 – Gibco), and 50U/mL of penicillin and streptomycin (15070063 – Gibco).

The human T lymphotropic virus 1 (HTLV-1) productively-infected SLB1 (RRID: CVCL_RT63) and HUT-102 (RRID: CVCL_3526) cell lines were cultured in RPMI 1640 medium (52400–025 – Gibco) supplemented with 50 U/ml of penicillin and streptomycin (15070063 – Gibco), with 20% FBS for the SLB1 cell line and 10% FBS (10270106 – Gibco) for the HUT-102 cell line.

The human lung carcinoma cell line A549 (CCL-185 – ATCC) was cultured in DMEM (11965092 – Gibco) supplemented with 10% FBS (10270106 – Gibco). These cells were infected with the human Adenovirus C5 (hAdV C5) (accession number: AC_000008.1; VR-5 – ATCC) as described in [30].

Lymphoblastoid cell lines (LCLs) were derived from calves developing malignant catarrhal fever (MCF) upon infection with strain C500 of alcelaphine gammaherpesvirus 1 (AlHV-1; accession number: KX905135.1) [31,32]. The virus was propagated in bovine turbinate (BT) fibroblasts (CRL-1390 – ATCC) and maintained by limited passage (<5) before calf infection.

For the supplemental data and figures, we also explored different viral strains, namely: (a) Influenza A virus (IAV) (accession numbers: MN220691- MN220698, data: SRR15305018 [33]); (b) Avian leukosis virus (ALV) (accession number: NC_001408, data: SRR7719537 [34]); (c) Japanese encephalitis virus (JEV) (accession number: AF045551.2, data: SRR11425577 [35]); (d) Human T-lymphotropic virus (accession number: NC_001436.1); (e) Hepatitis C virus (HCV) (accession number: AB047639, data: SRR27696424 [11]).

### Simulation of circRNA reads for the evaluation of sensitivity and specificity

Two random locations on the genome were selected to extract a subsequence that would serve as the basis of the reads. The splicing direction (forward or backsplicing) was chosen and the splicing sites extracted accordingly. Between 1 and 30 reads composed of 150 nucleotides were extracted spanning the splice junction with at least one nucleotide on each splice site. The read ID was attributed as follows [splicing direction]_[splice_junction]_[number_from_1_to_30]. The reads with IDs starting with “backsplice_junction” were considered as true positives; others as true negatives.

We shuffled these reads into a dataset of 1 000 000 random reads and 300 000 reads extracted from each host transcriptome (100 000 reads per host; human, cow and chicken). For U2 canonical simulated transcripts, we made sure to select backspliced reads that were surrounded by the “GT/AG” dinucleotides.

### RNA extraction and library preparation

RNAs were extracted from infected cells using TRI Reagent (AM9738 – Invitrogen), DNase I-treated as described in [16] and purified using RNA Clean and Concentrator-5 (R1016 – Zymo Research). The library was then prepared by Novogene (Cambridge, UK), following their circRNA sequencing protocol. It includes 1) a circRNA enrichment by depletion of ribosomal and other linear RNAs through RNase R treatment; 2) a fragmentation of the circRNAs; 3) first-strand cDNA synthesis using random hexamers before the proper strand-specific library preparation.

### Data cleaning and bioinformatics analysis

To clean up the reads, we discarded the unmapped reads after mapping on the viral genome with the Burrows Wheeler Aligner (BWA) [36] under the command “bwa mem -a -T15 [fasta reference file] [fastq sequencing file]”. Next, all recovered RNA-seq data were cleaned using Trimmomatic [37] following the author’s protocol. It required trimming the adapters, eliminating very short reads (shorter than 70 nucleotides) and filtering bad quality reads (Sliding Window 4:15). The duplicated sequences resulting from PCR artifacts were removed using the shell script dedupe.sh [38].

vCircTrappist is a pipeline relying on existing programs such as Samtools [39] and BWA followed by successive python scripts launched under a bash shell script. All the codes are available on the GitHub page of vCircTrappist (https://github.com/achasseu/vCircTrappist).

**Fig 1:**
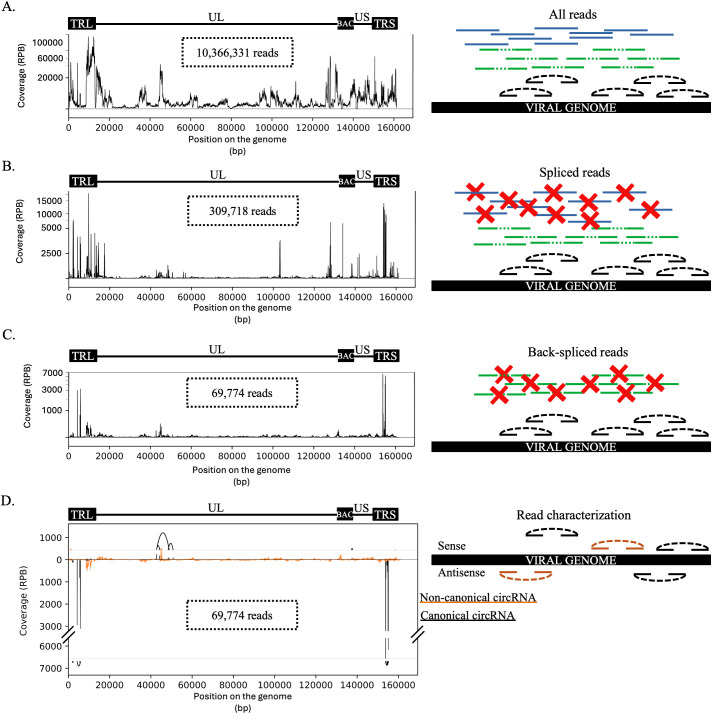
Description of the vCircTrappist process. (A) Mapping on the viral genome. The whole set of reads is mapped to the viral genome using BWA. (B) Sorting the spliced reads. The reads that present a splicing signature were recovered while the others were deleted. (C) Sorting the backsplice junction spanning reads. (D) Read characterization. The reads were sorted according to their features such as their splicing pattern or the strand they were originating from. The reads that correspond to a canonical U2 splicing pattern are depicted in black, while the non-canonical splicing reads are depicted in orange. The curves above and below the graph represent the 20 most abundant circRNA candidates found in the dataset. (A-D) On the left panels, the genome structure of the viral bacmid used for the transfection is depicted above the coverage plots. The genome is composed of two terminal repeats. The internal repeats were removed by the insertion of the bacmid cassette. The unique regions, short and long, are depicted. The X axis indicates the position on the viral genome (bp). The Y axis represent the normalized coverage. The normalization was made on the number of reads mapped at a specific locus divided by the total number of reads mapping on the viral genome, multiplied by one billion. On the right panels, the genome is depicted in black while the reads are depicted in green (linear splicing reads), blue (non-spliced reads) and black or orange (canonical and non-canonical backsplice junction spanning reads, respectively).

The first step (Fig 1A) of the pipeline is the alignment on the viral genome using BWA with the options “mem -a -T15”. The second step, run with splitfilter.py (Fig 1B), aims to isolate reads that exhibit splicing, removing those without at least one secondary alignment. The script analyzes flags in the alignment file, retaining reads that share the same ID, have one flag value at 2048 (or 2064 for reverse complement), and the other at 0 (or 16 for reverse complement). At this stage, reads mapping multiple times at various positions (flags 256 or 272 for reverse complement) are excluded. To avoid false negative results from multiple alignments in large viral genomes, we removed long (>5000 bp) repetitive elements such as herpesviral long terminal repeats.

The third step (Fig 1C), aiming at recovering the circRNAs, is carried out by several scripts, namely: circChaser.py, bsj_id.py, repet.py, circ_listing.py and circ_caracterisator.py. circChaser.py eliminates the reads originating from linear splicing. It analyzes the positions of reads with the same ID, identifying circRNA signatures. Specifically, if the start of a read is at position X, the end of the same read should be at position X-Y if it matches the sense strand, Y being an integer higher than 135 and smaller than 100,000. If it matches the antisense strand, the end of the read should be at position X + Y. This script produces a list of BSJ with their associated donor and acceptor backsplice sites (BSS).

bsj_id.py and repet.py, compare all BSJ and BSS to count backsplicing events. A backsplicing event is considered if two mapping reads have more than 95% identity conserved in their BSS sequences. During this analysis, donor and acceptor BSS of each event are compared to eliminate potential PCR or misalignment artifacts. Stretches of unique nucleotides or repetitive sequences like telomeric repeats, are removed at this step. Backsplicing events occurring more than once undergo a conclusive analysis with circ_listing.py and circ_caracterisator.py, characterizing them by identifying specific GT-AG motifs, which correspond to canonical splicing, at the BSS locations. The proximity of these sequences to the BSS attributes a score, which we utilize to categorize circRNAs as either canonical or non-canonical. For each BSS, a score of 0.5 is attributed if a “GU” (donor site, “AC” for reverse complement) or “AG” (acceptor site, “CU” for reverse complement) is located precisely at the splicing site. These sequences were chosen because they are conserved in the canonical splicing context. For each nucleotide between the splicing site and the nearest GU or AG sequence, the score is reduced of 0.1. Then the sum of the donor and acceptor scores (<= 1) is stored. Subsequently, this information is processed to generate four alignment files classifying backsplicing events based on their canonical status (score > 0.6) and further segregating them according to strand specificity.

These alignments are subsequently compiled using Samtools with the instructions “depth -a [FILENAME]” to generate a coverage table and Matplotlib to generate coverage plots and circRNA plots with covvisualisator.py and circ_visualisator.py (Fig 1D).

For the comparison with CIRI2, we followed the instructions provided by the publisher. This involved mapping the reads with BWA using the options “mem -a -T18” and utilizing both files of the paired-end sequencing. Subsequently, the resulting table was used to retrieve the circRNA reads from the alignment files. Samtools and a Matplotlib subscript (included in the vCircTrappist GitHub) were then employed to generate the coverage plots.

### RT-PCR confirmation

Reverse transcription was performed using the SuperScript IV kit (18090010 – Invitrogen) with random (250 nM; S1330S – New England Biolabs) or specific (100nM; Eurogentec) primers following the manufacturer’s recommendations. For each sample, a non-RT negative control was produced.

The PCRs were carried out using primers detailed in S1 Table and the GoTaq G2 kit (M7841 – Promega) or the Q5 taq polymerase (M0491L – New England Biolabs). The cycles were adapted to the manufacturers’ instructions. All the positive PCRs were purified using the Nucleospin Gel and PCR Clean-Up kit (740609 – Macherey-Nagel), cloned into a pGEM-T easy vector using T4 DNA ligase (A1360 – Promega) using the TG1 strain of *E.coli*. Clones were sequenced by Sanger sequencing (Eurofins Genomics) for sequence confirmation.

### Grammatical and phrasing corrections

ChatGPT 3.5 (OpenAI) was used for grammatical purposes. No data, interpretation or texts were generated *de novo* using this tool.

## Results

### vCircTrappist: A bioinformatics pipeline for viral circRNA discovery

In our effort to identify novel circular RNAs (circRNAs), we initially relied on widely used circRNA detection programs. However, we observed significant discrepancies between their predictions and our PCR validation results [16]. These inconsistencies prompted us to develop vCircTrappist, a dedicated pipeline specifically designed to comprehensively detect circRNA candidates expressed by viruses during their infectious cycle independently of their GU-AG backsplicing signature.

The vCircTrappist pipeline focuses on the identification of backsplicing signals (Fig 1). As a first step, raw sequencing data were preprocessed and the viral genome prepared for circRNA identification. For the Marek’s Disease Virus (MDV), we removed large genomic duplications that could interfere with accurate read mapping, while retaining smaller repetitive elements. To test and develop vCircTrappist, we analyzed high-throughput sequencing data obtained from an *in vitro* productive infection of the ESCDL-1 cell line by MDV. Following sequencing, we generated a dataset of 10,366,331 reads mapping to the MDV genome (Fig 1A).

Following this first alignment, we filtered for spliced reads, independent of splicing type. Only reads mapping uniquely to a single viral genome segment and exhibiting at least one secondary alignment were retained. Reads mapping to repetitive regions, frequent in viral genomes, identified by the presence of multiple primary alignments, were discarded. This filtering step reduced the dataset to 309,718 reads (Fig 1B).

We then examined the mapping positions of primary and secondary alignments to identify backsplicing signatures (Fig 1C). Reads showing a secondary alignment upstream in the genome sequence, while this alignment appeared downstream in the read sequence, were considered as spanning a backsplicing junction (BSJ). These reads were retained along with their backsplicing site (BSS) and BSJ coordinates for downstream analysis. Additional filters were applied to exclude reads containing large repetitive elements, such as long homopolymeric stretches or telomeric repeats.

After filtering, we grouped the remaining reads based on the position of their splicing sites to quantify distinct junctions. Junctions supported by multiple reads were prioritized as likely genuine circRNA candidates. After applying all filters and selection steps, we recovered a total of 69,774 circRNA-supporting reads (Fig 1C).

The final dataset was further sorted based on strand orientation and backsplicing type (Fig 1D). Junctions that followed the canonical GT-AG splice motif were classified as canonical U2, while those not following this pattern were considered as non-canonical splicing events. The vCircTrappist pipeline outputs a summary table (S2 Table) detailing the identified circRNAs, including the sequence and positions of their splicing sites, read counts, splicing pattern, predicted circRNA size, gene locus, the longest common sequence between splicing sites (to minimize false positives), the viral genome reference, and the identifiers of the supporting reads. The pipeline also generates publication-ready visualizations of the circRNA-supporting reads (Fig 1D).

Altogether, vCircTrappist offers a robust and biologically informed approach for the identification of viral circRNAs. Its ability to account for viral genome-specific features, including repetitive elements, compact transcriptional architecture, and non-canonical splicing, makes it a valuable tool for circRNA discovery in the context of viral infections.

### vCircTrappist is a robust tool to identify non-canonical circRNAs

**Fig 2:**
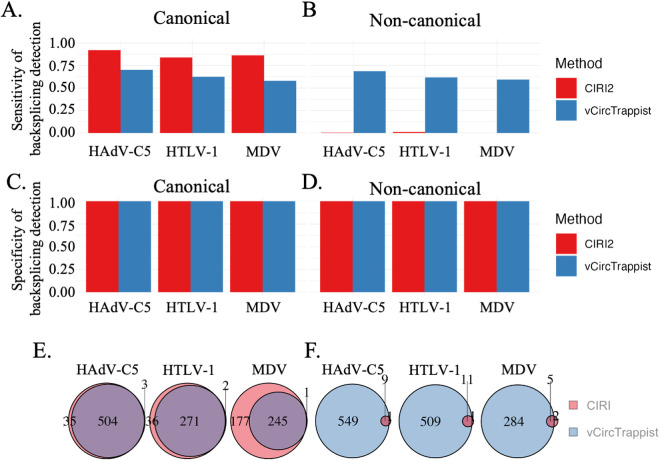
*In silico* comparison of vCircTrappist with CIRI2. (A, B) Sensitivity (true positive rate) of the detection of canonical (A) or non-canonical (B) backsplicing events from reads originating from indicated viral genomes. (C, D) Specificity (1 - false positive rate) of the detection of canonical (C) or non-canonical (D) backsplicing events from reads originating from indicated viral genomes. (E, F) Venn diagrams indicating the number of specific or common unique canonical (E) or non-canonical (F) backsplice junctions by CIRI2 and vCircTrappist from reads originating from indicated viral genomes.

To validate the efficacy of vCircTrappist in viral circRNA identification, we compared its performance with the widely employed program, CIRI2 [19] (Fig 2). Given the absence of a gold standard for circRNA identification from RNA-seq data, our initial strategy to assess the sensitivity and specificity of vCircTrappist was to generate simulated datasets, from which we could precisely quantify the discovery rates. To ensure vCircTrappist’s applicability across diverse viral families, we used three different infection models: an herpesvirus, the Marek’s disease virus (MDV); an adenovirus, the human adenovirus C5 (hAdV-C5); and a retrovirus, the human T-lymphotropic virus 1 (HTLV-1). To monitor the sensitivity and specificity of both programs, we generated datasets based on two different scenarios: the first one representing canonical U2 backsplicing (Fig 2A; 2C; 2E) and the second one representing random canonical and non-canonical backsplicing events (Fig 2B; 2D; 2F).

We simulated RNA-seq experiments for the viral infections as follows: we first generated one million random 150-base sequences to mimic background noise. We then added 300,000 reads simulating host linear RNA-seq (from human, cow, and chicken; our infection models). To introduce controlled circular events, we randomly generated between 500 and 1500 forward or back-splice junctions in the viral genomes and created between 1 and 30 reads mapping to each of these junctions. Each read was flagged as either “true positive” or “true negative” for subsequent benchmarking. Finally, we shuffled all the reads to simulate a realistic dataset.

Using vCircTrappist and CIRI2, we aimed to recover the “true positive”-flagged reads to evaluate both tools’ sensitivity and specificity. In a first trial, we only focused on canonical backsplicing events (Fig 2A). In that scenario, both CIRI2 and vCircTrappist achieved to display a specificity of 100% (Fig 2C). In terms of sensitivity, CIRI2 was more robust with a sensitivity >83% compared to 58–68% for vCircTrappist for all three viruses (Fig 2A). In the second scenario (Fig 2B), for which the reads were generated based on random backsplicing events, CIRI2’s sensitivity dropped dramatically to <2%, while vCircTrappist demonstrated a stable value of >55%, without sacrificing specificity (False Discovery Rate, FDR = 0) (Fig 2D).

Since both pipelines have different abilities to identify circRNAs depending on their splicing pattern, we wondered if the candidates identified by these pipelines were common or not. Therefore, we compared the lists of identified backspliced junctions by each program and counted the intersecting elements (Fig 2E, 2F). In the canonical splicing population, most reads identified by vCircTrappist were identified by CIRI2 (Fig 2E). The situation was reversed when looking at non-canonical splicing, for which only one or two backsplice junctions identified by CIRI2 were not detected by vCircTrappist (Fig 2F).

### vCircTrappist reveals more circRNA candidates than CIRI2 from viral infections datasets

Since we proved the usability of vCircTrappist for detecting non-canonical splicing, we proceeded with an analysis of RNA-seq data obtained from *in vitro* infection models. It is noteworthy that visualization of the CIRI2 results required the development of a Matplotlib subscript to provide graphical representation of BSJ reads. This subscript is available on the vCircTrappist GitHub page.

**Fig 3:**
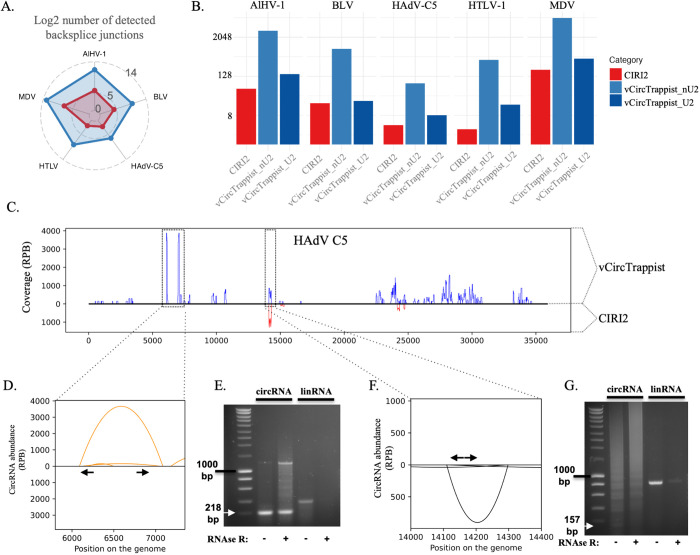
*In vitro* comparison of vCircTrappist with CIRI2. (A) Number of backsplice junctions discovered by vCircTrappist and CIRI2 from *in vitro* circRNA sequencing data. For each virus, the raw log2 number of unique backsplice junctions was measured. The count obtained using vCircTrappist is depicted in blue, while the count obtained using CIRI2 is depicted in red. (B) Comparison of vCircTrappist vs CIRI2 in multiple infection contexts. The barplots represent the number of identified unique backsplice junctions for each program. For vCircTrappist, we segregated the canonical from non-canonical backsplice junctions. (C) vCircTrappist vs CIRI2 coverage of circRNA reads on the HAdV C5 genome. vCircTrappist reads are mapped to the upper part of the graph, while CIRI2 reads are mapped below. The Y axis represents the coverage in circRNA reads per billion reads mapped to the viral genome. The X axis represents the positions on the genome in base pairs. (D; F) Analysis of the coverage obtained with vCircTrappist. The Y axis indicates the number of times individual reads have mapped to a specific backsplice junction. The X axis indicates the positions (in bp) where the circRNA are backspliced. The positions of the primers for subsequent RT-PCR confirmation are indicated by black arrows. (E; G) PCR confirmation of the loci that were identified by vCircTrappist. White arrows were placed at the expected band size to facilitate reading. The smears indicate the presence of alternative circRNA transcripts. The ladder is the SmartLadder from Eurogentec, the 1kb band is indicated on the Figure.

Using the same viral strains as in the simulations along with the bovine leukemia virus (BLV) and the alcelaphine herpesvirus 1 (AlHV-1), we sequenced circRNAs from productive *in vitro* infections and compared the results obtained with vCircTrappist and CIRI2 (Fig 3A). In all infection contexts, vCircTrappist outperformed CIRI2 in terms of the number of detected backsplice junctions. Upon closer examination (Fig 3B), we observed that CIRI2 revealed comparable numbers of identified backsplice junctions when we only accounted for canonical splicing in vCircTrappist data. We thus conclude that while both CIRI2 and vCircTrappist are effective for detecting canonical circRNAs in biological data, vCircTrappist is more suited for identifying non-canonical splicing events. As supported by previous studies [11,13,40,41], non-canonical splicing mechanisms may be widely exploited by viruses, positioning vCircTrappist as a valuable tool to uncover these unique transcripts.

To further support our findings, we next focused on the adenovirus infection model, which showed the highest circRNA detection rates when using both vCircTrappist and CIRI2 (Fig 2). Comparative coverage plots from both pipelines (Fig 3C) revealed numerous circRNAs uniquely identified using vCircTrappist. Interestingly, both pipelines detected a highly abundant circRNA, which CIRI2 appeared to quantify at a higher level than vCircTrappist. This circRNA was confirmed to arise from canonical splicing. We attribute the observed abundance difference to a key pipeline distinction: vCircTrappist analyzes only one mate from paired-end sequencing, while CIRI2 processes both. We deliberately designed vCircTrappist this way because circRNA rolling-circle amplification during reverse transcription can generate concatemers, leading to potential biases in paired-end sequencing, as both mates may map to the same BSJ [11]. This amplification may bias subsequent analyses when using paired-end sequencing, as both ends of the reads might map to the same BSJ.

We then focused on two viral genomic regions involved in viral pathogenesis where vCircTrappist identified circRNA candidates, one originating from non-canonical splicing (Fig 3D) and one originating from canonical splicing (Fig 3F). We designed divergent PCR primers for both loci and successfully amplified both targets by RT-PCR before and after RNase R treatment (Fig 3E; 3G). RNase R is an exonuclease able to digest linear RNAs but not circRNAs, supporting the biological relevance of vCircTrappist’s predictions.

Collectively, our results highlight the vast wealth of information provided by vCircTrappist when compared to CIRI2, the current bioinformatics pipeline most frequently used for the identification of viral circRNAs. Notably, the circRNAs newly identified using vCircTrappist are linked to genes implicated in viral pathogenesis, further emphasizing vCircTrappist’s potential to shed light on crucial circular transcripts.

### vCircTrappist uncovers novel viral circRNAs

**Fig 4:**
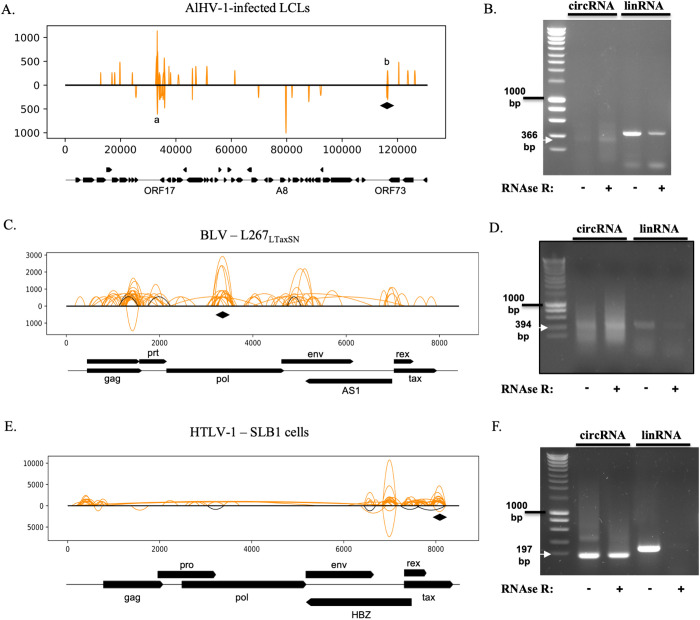
Investigation of unexplored viral circRNAs from diverse infections. (A) Viral circRNAs expressed from an *in vivo* infection with AlHV-1. A lymphoblastoid cell line (LCL) from *in vivo*-infected calves was recovered and grown in culture. (B) PCR confirmation of the results obtained by Illumina Sequencing. (C) Viral circRNAs expressed from BLV productively-infected cell line L267_LTaxSN_. (D) PCR confirmation of the results obtained by Illumina Sequencing. (E) Viral circRNAs expressed from an *in vitro* culture of productively HTLV-1-infected cells (SLB1 cell line). (F) PCR confirmation of the results obtained by Illumina Sequencing. (A,C,E) The 100 more expressed circRNAs are depicted on the graphs. The X axis represents the position on the viral genome. The Y axis indicates the number of times individual reads have mapped to a specific BSJ divided by the total number of reads mapping on the viral genome and then multiplied by one billion. The black lines represent the canonical circRNAs. The orange lines represent the non-canonical circRNAs. The height of the curves represents the normalized abundance of each individual backsplice junction. The lines up from the genome are mapped in the same sense than the viral genome, while the lines under are antisense to the viral genome. The relevant ORFs of the viral genome are depicted under the graphs. For graphical purposes, the splicings of the tax and rex genes were not depicted. The loci targeted for RT-PCR confirmation are indicated by black losanges. Peaks of interest described in the text are indicated by lowercase letters. (B,D,F) Every sample was treated with RNase R before RT to enrich circRNAs in the sample. For each circRNA candidate, a convergent PCR control was carried out to ensure the proper degradation of linear RNAs in the sample. The ladder is the SmartLadder from Eurogentec; the 1kb band is indicated next to the gel picture. White arrows were placed at the expected band size to facilitate reading.

Having established the competence of vCircTrappist for the discovery of novel viral circRNAs, we proceeded to apply this pipeline to both new (Figs 4, S2–S4) and existing datasets (Figs S1, S5–S6). In this comprehensive investigation, we delved into the realm of circRNAs expressed by a wide spectrum of DNA and RNA viruses.

We applied vCircTrappist to multiple infection contexts. Notably, we explored circRNA profiles in two animal and one human models associated with virus-induced lymphoproliferation: AlHV-1, HTLV-1 and BLV (Figs 4A–4F and S2). In these three viral infection contexts, vCircTrappist identified a multitude of new circRNA candidates. To validate these findings, we confirmed circRNAs for each virus via divergent RT-PCR and included RNase R controls. We also included RNase R-non-treated controls to monitor the efficiency of the treatment in purifying circRNAs (Figs 4B; 4D; 4F).

The AlHV-1 infection revealed abundant circRNA expression through vCircTrappist analysis. We unveiled numerous circRNAs loci and one of them was subsequently confirmed through RT-PCR (Fig 4A, 4B). One striking discovery was a locus (labeled “a” in Fig 4A) previously unassociated with any known transcript or open reading frame (ORF). While the circRNAs associated with this locus did not adhere to a canonical U2 splicing pattern, the peak signature in the coverage plot revealed conserved profile observed from two independent *in vivo* samples. This suggests that this highly expressed locus might be the source of non-coding circRNAs possibly involved in virus-induced pathogenesis. A second locus (labeled “b” in Fig 4A) is linked to the expression of the ORF73 protein, responsible for maintaining the episomal viral genome within the infected cell [31,42]. Collectively, our results obtained from AlHV-1 infection highlighted the production of circRNAs of viral origin in another viral induced lymphoproliferative disease.

In the context of a retroviral infection, similar results were obtained by analyzing the viral circRNAome. Three different models of retroviral infection by BLV were used: two of these were tumor-derived BLV-infected cell lines constitutively producing high levels of viral transcripts (YR2_LTaxSN_ and L267_LTaxSN_), while the third one was an *in vitro* model of BLV infection generated by transfection of human-derived uninfected 293T cells with a plasmid expressing a complete and infectious BLV clone. The obtained data presented intriguing features as they lacked a specific splicing pattern and conserved splicing sites (Figs 4C and S2). However, they exhibited exceptional reproducibility in three distinct models of productive viral infection (S2 Fig). Data obtained before reactivation did not reveal any circRNA expression during latency, most probably due to a reduced level of viral transcripts. Nevertheless, the consistency across the three sequencing results from productive infections established a key point: the generation of circRNAs was not contingent on the cell type. The major circRNA expression peak was located in the *pol* gene, which encodes for the viral reverse transcriptase. Utilizing divergent PCR, we confirmed the presence of circRNAs originating from this region in a relevant infection model (L267_LTaxSN_) (Figs 4D and S7K).

We also carried out a circRNA sequencing on Human T Lymphotropic Virus 1 (HTLV-1)-infected cell lines (Figs 4E, 4F and S3) since BLV stands as a robust animal model for this related human retrovirus. This revealed strong coverage peaks in the 3’ end of the HTLV-1 genome in the vicinity of the *tax* and *rex* genes. The Tax protein is responsible for the reactivation of the HTLV-1 transcription and replication cycle.

In all these infection contexts, we were able to obtain multiple PCR amplicons (Figs 4B; 4D; 4F and S7). Two elements in these gels confirmed the circular shape of these RNAs. The first was the observation of the RT-induced rolling circle amplification of circRNAs as discussed in the previous paragraph. It was mostly observed with abundant circRNAs (S7P Fig). In this case, the sizes of the amplicons corresponded to the size of the initial amplicon added to multiple times the size of the circRNA. The other confirmation was the resistance of these circRNAs to the exonuclease activity of RNase R shown in all the displayed gels and in the vCircTrappist HCV results (S1 Fig). While this was again a strong proof-of-concept for vCircTrappist to be able to identify accurately circRNAs, it also led to the discovery of a candidate sensitive to RNaseR treatment, likely a false positive (S7J Fig).

We also investigated four viral circRNAome using preexisting datasets (S5 and S6 Figs). In all cases we identified numerous circRNA candidates. More precisely, we were able to identify 1023 unique BSJs for the Influenza A virus (IAV) (S5 Fig, Table 1), while we obtained 135 for the Avian Leukosis virus (ALV) (S6A Fig, Table 1), 2764 for Japanese Encephalitis Virus (JEV) (S6B Fig, Table 1) and more than 135 for HCV (S1 Fig, Table 1). While we could not address the roles of such an amount of circRNA candidates, we wondered if they might serve to increase the coding potential of viruses. Focusing on ORFs longer than 450 nucleotides that span backjunctions, we identified numerous potential alternative proteins that could be encoded from circular RNAs (Table 1). More experiments, including ribosome profiling or reporter gene assays will be necessary to assess their coding potential.

**Table 1:** Amount of unique circRNA candidates identified for each virus and potential associated alternative ORFs. The samples marked by an asterisk were acquired in the context of the present study.

Virus	Number of unique backsplice junctions	Number of alternative ORFs (>150aa)
Marek’s disease virus (MDV)*	8453	1530
Alcelaphine Herpesvirus 1 (AlHV-1) - Cow 1*	302	22
Alcelaphine Herpesvirus 1 (AlHV-1) - Cow 2*	3410	352
Human Adenovirus C5 (HAdV C5) – 12h p.i.*	13	0
Human Adenovirus C5 (HAdV C5) – 18h p.i.*	86	8
Human Adenovirus C5 (HAdV C5) – 24h p.i.*	159	13
Influenza A virus (IAV) – SRR15305018	1023	105
Avian Leukosis Virus (ALV) – SRR7719537	135	8
Japanese Encephalitis Virus (JEV) – SRR11425577	2764	1119
Bovine Leukemia Virus (BLV) - YR2_LTaxSN_*	918	152
Bovine Leukemia Virus (BLV) – transfected 293T*	768	95
Bovine Leukemia Virus (BLV) – L267_LTaxSN_*	471	102
Human T Lymphotropic Virus 1 (HTLV-1) – HUT102*	147	16
Human T Lymphotropic Virus 1 (HTLV-1) – SLB1*	427	43
Hepatitis C Virus (HCV) - SRR27696424	136	27
Hepatitis C Virus (HCV) - SRR27696425	159	30

Collectively, these results demonstrate vCircTrappist as an exemplary tool for the identification of viral circRNAs within diverse infectious contexts.

## Discussion

In this study, we have introduced vCircTrappist, a novel bioinformatics pipeline meticulously designed for the comprehensive exploration of circularization events within viral transcripts. By imposing rigorous filters, we successfully isolated these distinct sequences from both new and existing datasets. When compared to CIRI2, the current gold standard for circRNA identification, our vCircTrappist program demonstrated superior sensitivity and robustness in detecting non-canonical circRNA candidates with potential implications in the pathogenesis process. Therefore, these loci deserve further characterization. Although other programs have been assessed [20,21], they were not evaluated in our analysis as they exhibit similar limitations to CIRI2 in the context of viral circRNAs. Notably, the fact that 1) they are based on paired-end sequencing that tends to overestimate the abundance of circRNAs, and 2) they all rely on the recognition of canonical splicing patterns. Our findings, particularly from adenoviral, herpesviral and retroviral infections, are of significant note, underlining the potential of vCircTrappist to become the future gold standard for viral circRNA identification.

Our results offer promise in the development of an open, unbiased, and biologically relevant approach for viral circRNA discovery. While the concept of identifying BSJ-covering reads is not new [19–21], the recent characterization of numerous non-canonical circRNAs [10,12–14,16] necessitates a departure from the stringent parameters imposed by current programs. In our comparative analysis, vCircTrappist consistently found more hits than CIRI2 across various viral infection contexts. While CIRI2 is acknowledged as the gold standard for cellular circRNAs, only vCircTrappist demonstrated precise mapping of numerous non-canonical circRNAs on viral genomes. This enhanced detection can be attributed to our choice of employing diverse criteria for circRNA identification, applying custom filters tailored to the unique attributes of viral genomes. For example, we deliberately excluded reads spanning repeated regions to eliminate potential inaccuracies. This is because many viruses encompass large repeated sequences in their genome for regulatory purposes. In this context, we can cite the Long Terminal Repeats (LTR) in retroviruses or the diverse repeat regions in herpesviruses. However, it is crucial to note that these repeated regions may serve as sources of circRNAs, and with current technologies, distinguishing them from linear RNAs is challenging. In our case, to study genes located in the inverted repeats of MDV, we kept only one of these copies and suggest the reader to proceed in the same manner.

Our newfound data concerning four distinct viruses (AlHV-1; BLV; HTLV-1; HAdV C5) provides valuable insights into the strengths and potential limitations of vCircTrappist thanks to numerous RT-PCR confirmations of circRNA candidates. During our investigation of HAdV C5 circRNAs, we encountered a circRNA candidate that, upon further analysis, was determined to be an intron-derived transcript. Yet, its expression pattern (S4 Fig) suggested that the transcript was stably expressed throughout the infection course, which, in itself, was intriguing and worthy of further investigation. This finding illustrated that vCircTrappist could identify transcripts beyond traditional circRNAs, highlighting its potential for serendipitous discoveries.

In both BLV and HAdV C5 data, we observed circRNAs expressed in the vicinity of the *pol* genes. While these two genes use different mechanisms to generate new viral (pro)genomes, these observations might reflect the role of circRNAs in genome maintenance and/or duplication. We hypothesize that the circRNAs produced from these spots might interact with the proteins produced from the same genes and alter the processing of the nascent (pro)genome. However, we were not able to confirm this hypothesis, and further work will be required to investigate this issue.

The case of AlHV-1 also warrants attention. While the identification of abundant non-coding transcripts during a herpesviral infection is not unexpected [16,41,43], the primary expression peak is positioned at a locus that has not been previously characterized, as it is not associated with any ORF. However, it is in close proximity to the expression locus of the viral microRNAs (miRNAs). Although this locus is not linked to the pathogenesis of AlHV-1 [32], it may act concomitantly with the newly described circRNAs or, conversely, mitigate their effect. Previous research on the Epstein-Barr Virus (EBV) [9] has shown that the circRNA associated with the Latent Membrane Protein 2A sponges a miRNA involved in p53 regulation, a regulatory cycle linked to the progression of gastric cancer. Another possibility is the involvement of these circRNAs as non-coding transcripts essential for the establishment of latency, as demonstrated for diverse alphaherpesviruses [44–46]. Conversely, Ungerleider [43,47] proposed a mechanism in which circRNAs may help maintain the herpesviral lytic cycle by interacting with the viral origin of replication. This region has not yet been described in AlHV-1 and may correspond to one of the identified circRNA hotspots. In summary, the discovery of circRNAs produced during AlHV-1-induced lymphoproliferation may offer a fresh perspective on viral pathogenesis.

Overall, when we looked at all the viral circRNA candidates, we also demonstrated the high number of putative ORFs in the vicinity of circRNAs. We were not able to demonstrate their usability in the context of this study but other examples of viral coding circRNAs exist. The HPV-derived circE7 was shown to have a prominent role in virus-induced oncogenesis and several papers describe the impact of circRNA-derived micropeptides on the viral cycle of reoviruses [7,15,48]. We suggest that such roles might be more widespread in viruses. Creating a wide range of different ORFs, leading to a wide range of different peptide decoys might also be a way to avoid the immune response, by competing with immune-relevant peptides for MHC presentation.

Our extensive analysis of diverse viruses highlights the potential impact of vCircTrappist on the virology community. Along with previous studies [10,12–14,16,17,22], we once again observe that viral circRNAs are primarily produced in non-canonical ways. It is worth noting that this mechanism might be more universal and could involve both viral and cellular factors, as non-canonical circRNAs have also been identified in plant and metazoan non-infected cells [49,50]. While we cannot provide a definitive mechanistic explanation for this phenomenon, previous research [17] has identified the involvement of the exonuclease activity of the NSP14 protein in the generation of these transcripts during a coronavirus infection. This research did not speculate on the mechanism involved behind this, but we suggest a hypothesis in which circRNAs might be generated due to collision-induced pauses during transcription. This hypothesis relies on the fact that viruses tend to transcribe faster than their host [51]. In that case, one could imagine that viral genomes might be covered by RNA polymerases that would collide when encountering obstacles. These obstacles might be of several natures: RNA structures, viral or immune-related proteins,… Thus, it would induce a pause in transcription and it is possible that the energy required for the RNA elongation may be inadvertently redirected into binding both extremities of a linear RNA molecule. Unfortunately, no data exist yet on such a phenomenon and the recency of the discovery of non-canonical RNAs makes it hard to find and elucidate such hypotheses.

In summary, we present here a pioneering bioinformatics pipeline with the potential to unveil previously uncharted roles within viral transcripts characterized by diverse splicing patterns. Altogether these analyses allowed us to detect an important number of unique BSJ from various viral infection models as presented in Table 1. The study opens the door to an intricate field that needs thorough exploration. It began with the description of new circRNAs from *in vitro* and *in vivo* infections (Figs 2 and 3). Then we analyzed their production during an infection kinetics (S4 Fig). And finally, we explored existing datasets with the ALV, HCV and JEV (S1 and S6 Figs) and with the different segments of the IAV (S5 Fig). Nonetheless, the circRNA landscape of the vast majority of viruses remains to be explored.

## Supporting information

S1 Fig Impact of RNase R treatment on circRNA candidates identified by vCircTrappist during an HCV infection. Huh7 cells were infected with the JFH-1 strain of the virus in the context of the study of Cao *et al*, 2024 (11). (A) CircRNA identification by vCircTrappist before RNase R treatment. (B) CircRNA identification by vCircTrappist after RNase R treatment. The datasets represent a biological duplicate.
(TIF)

S2 Fig Viral circRNA identification in three models of BLV infections. (A) CircRNAs mapped to the BLV genome after a transfection of the viral genome in the HEK 293T cell line. (B) CircRNAs mapped to the BLV genome during a productive infection in the L267_LTaxSN_ cell line. (C) CircRNAs mapped to the BLV genome during a productive infection in the YR2_LTaxSN_ cell line. Relevant ORFs were indicated under the graphs on the same scale as the viral genome. For graphical purposes, the splicings of the *tax* and *rex* genes were not depicted. The Y axis indicates the abundance of unique circRNAs in reads mapping the backsplice junctions per billion of reads mapping on the viral BLV genome.
(TIF)

S3 Fig Viral circRNA identification in two HTLV-1 infection models. (A) CircRNAs mapped to the HTLV-1 genome of the productively-infected cell line HUT102. (B) CircRNAs mapped to the HTLV-1 genome of the productively-infected cell line SLB1. Relevant ORFs were indicated under the graphs on the same scale as the viral genome. For graphical purposes, the splicings of the *tax* and *rex* genes were not depicted. The Y axis indicates the abundance of unique circRNAs in reads mapping the backsplice junctions per billion of reads mapping on the HTLV-1 viral genome.
(TIF)

S4 Fig Viral circRNA identification during the course of an adenoviral infection. The circRNAs were mapped to the hAdV-C5 genome after 12h (A), 18h (B) or 24h (C) of infection. The infection was performed using A549 cells. Relevant ORFs were indicated under the graph on the same scale as the viral genome. The Y axis indicates the abundance of unique circRNAs in reads mapping the backsplice junctions per billion of reads mapping on the viral HAdV-C5 genome.
(TIF)

S5 Fig Viral circRNA identification from an IAV infection. (A-H) A549 cells were infected with the H1N1 strain of IAV in the study of Min *et al.*, 2023. The eight segments of the virus were processed through vCircTrappist and depicted in the Figure. The Y axis indicates the abundance of unique circRNAs in reads mapping the backsplice junctions per billion of reads mapping on the viral IAV H1N1 genome.
(TIF)

S6 Fig CircRNA identification in multiple viral infections. (A) Viral circRNAs identification from an ALV infection. The dataset was obtained after the infection of Chicken Embryonic Fibroblasts (CEF) in the study of Yang *et al.*, 2022 [34]. (B) Viral circRNAs identification from a JEV infection. Mouse brains were injected with the virus and harvested after 5 days of infection in the study of Li *et al*, 2020 [35]. The Y axis indicates the abundance of unique circRNAs in reads mapping the backsplice junctions per billion of reads mapping on the viral genome.
(TIF)

S7 Fig RT-PCR confirmations of the vCircTrappist results. (A) Viral circRNAs expressed from an *in vivo* infection with AlHV-1. A lymphoblastoid cell line (LCL) from *in vivo*-infected calves was recovered and grown in culture. (B-C-D) RT-PCR confirmations of the loci of circRNA expression in the AlHV-1 model. Untreated samples and corresponding linear RNAs were used as controls for the RNase R treatment. White arrows were placed at the expected band size to facilitate reading. (E) Viral circRNAs expressed from an *in vitro* infection with MDV. The ESCDL-1 cell line was infected with the RB-1B strain of the virus and the RNAs were recovered after 6 days of infection. (F-G) RT-PCR confirmations of the loci of circRNA expression in the MDV model. Untreated samples and corresponding linear RNAs were used as controls for the RNase R treatment. White arrows were placed at the expected band size to facilitate reading. (H) Viral circRNAs expressed from an *in vitro* culture of productively HTLV-1-infected cells (SLB1 cell line). (I-J) RT-PCR confirmations of the loci of circRNA expression in the HTLV-1 model. Untreated samples and corresponding linear RNAs were used as controls for the RNase R treatment. The second sample represents a false positive result. White arrows were placed at the expected band size to facilitate reading. (K) Viral circRNAs expressed from BLV productively-infected cell line L267_LTaxSN_. (L-M) RT-PCR confirmations of the loci of circRNA expression in the BLV model. Untreated samples and corresponding linear RNAs were used as controls for the RNase R treatment. White arrows were placed at the expected band size to facilitate reading. (N) Viral circRNA identification after 24h of an infection with the HAdV-C5. The infection was performed using A549 cells. (O-P) RT-PCR confirmations of the loci of circRNA expression in the HAdV-C5 model. Untreated samples and corresponding linear RNAs were used as controls for the RNase R treatment. White arrows were placed at the expected band size to facilitate reading. (A-E-H-N) Relevant ORFs were indicated under the graphs on the same scale as the viral genome. The Y axis indicates the abundance of unique circRNAs in reads mapping the backsplice junctions per billion of reads mapping to the viral genome.
(TIF)

S1 Table Primers used to confirm the vCircTrappist results. (XLSX)

S2 Table Example of dataset extracted using vCircTrappist. This example displays the data obtained for MDV, as in Figure 1 and S7E. The three first columns represent the backsplice junction and the backsplice sites. The « Count » column represent the raw number of reads mapping the backsplice junction without normalization. The Sense_score column is calculated based on the strand which is covered by the reads mapping the backsplice junction. The U2_score is calculated based on the distance of the backsplicing site from a canonical splicing site. The 5’ and 3’ position are the positions of the splicing sites and they determine the Max_CircRNA_Size. The « Colocalized Gene » column requires a completed GFF file to determine the colocalizing genes. The « Longest Common Kmer » was identified comparing the backsplice sites and the « Common Sequences » were extracted accordingly. The « Chromosome » is the viral genome reference on which the backsplice junction was identified. The identifiers of the reads are available in the SAM alignment file.
(CSV)
