# Supplementary figures and images for: Unbiased and comprehensive identification of virus-derived circular RNAs in a large range of viral species and families
Source: PLoS Pathog. 2025 Sep 11;21(9):e1013448. doi: 10.1371/journal.ppat.1013448 (PMC12425286; doi:10.1371/journal.ppat.1013448)

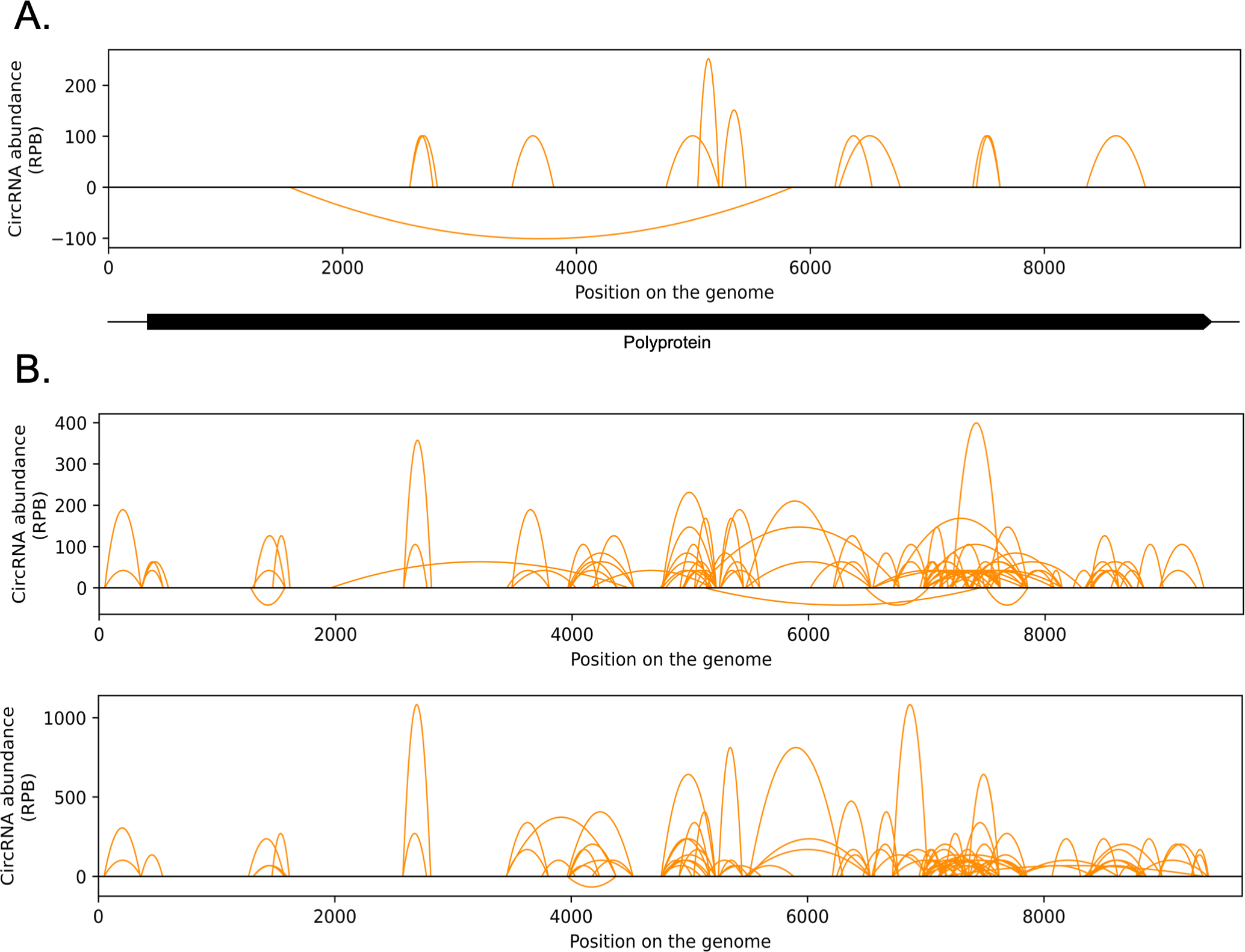

Supplement: S1 Fig — Huh7 cells were infected with the JFH-1 strain of the virus in the context of the study of Cao et al, 2024 (11). (A) CircRNA identification by vCircTrappist before RNase R treatment. (B) CircRNA identification by vCircTrappist after RNase R treatment. The datasets represent a biological duplicate. (TIF) [file ppat.1013448.s001.tif]

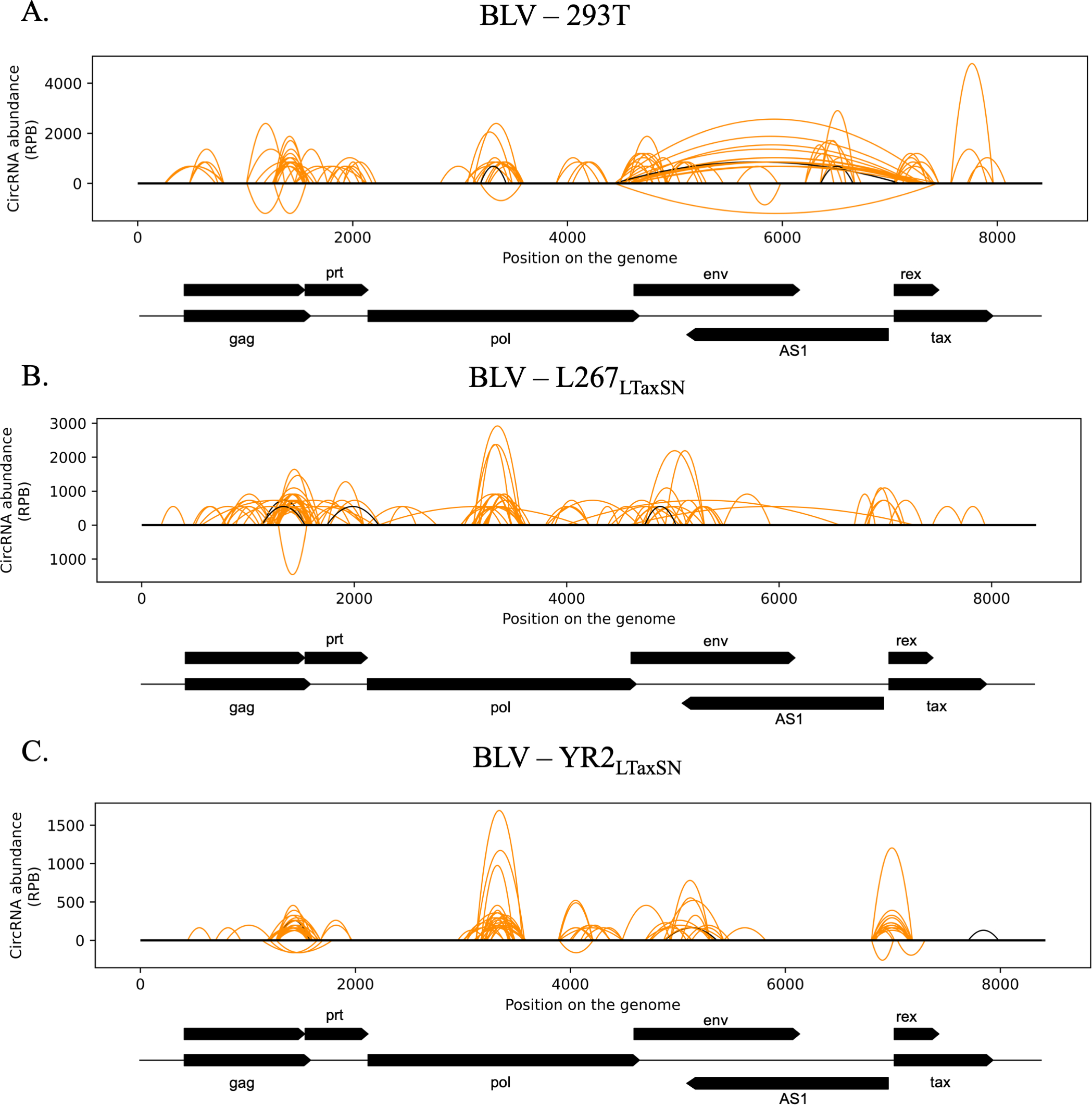

Supplement: S2 Fig — (A) CircRNAs mapped to the BLV genome after a transfection of the viral genome in the HEK 293T cell line. (B) CircRNAs mapped to the BLV genome during a productive infection in the L267LTaxSN cell line. (C) CircRNAs mapped to the BLV genome during a productive infection in the YR2LTaxSN cell line. Relevant ORFs were indicated under the graphs on the same scale as the viral genome. For graphical purposes, the splicings of the tax and rex genes were not depicted. The Y axis indicates the abundance of unique circRNAs in reads mapping the backsplice junctions per billion of reads mapping on the viral BLV genome. (TIF) [file ppat.1013448.s002.tif]

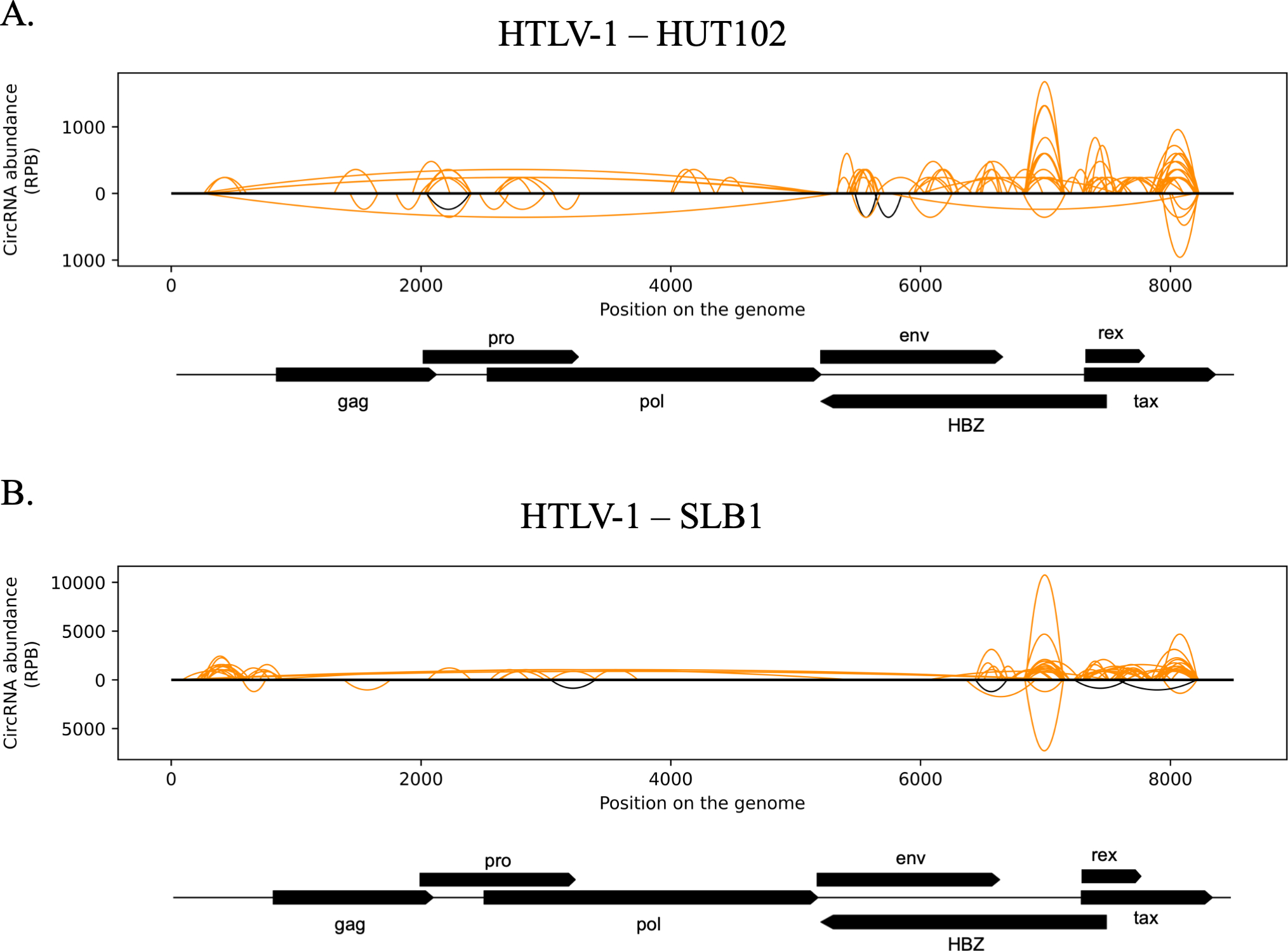

Supplement: S3 Fig — (A) CircRNAs mapped to the HTLV-1 genome of the productively-infected cell line HUT102. (B) CircRNAs mapped to the HTLV-1 genome of the productively-infected cell line SLB1. Relevant ORFs were indicated under the graphs on the same scale as the viral genome. For graphical purposes, the splicings of the tax and rex genes were not depicted. The Y axis indicates the abundance of unique circRNAs in reads mapping the backsplice junctions per billion of reads mapping on the HTLV-1 viral genome. (TIF) [file ppat.1013448.s003.tif]

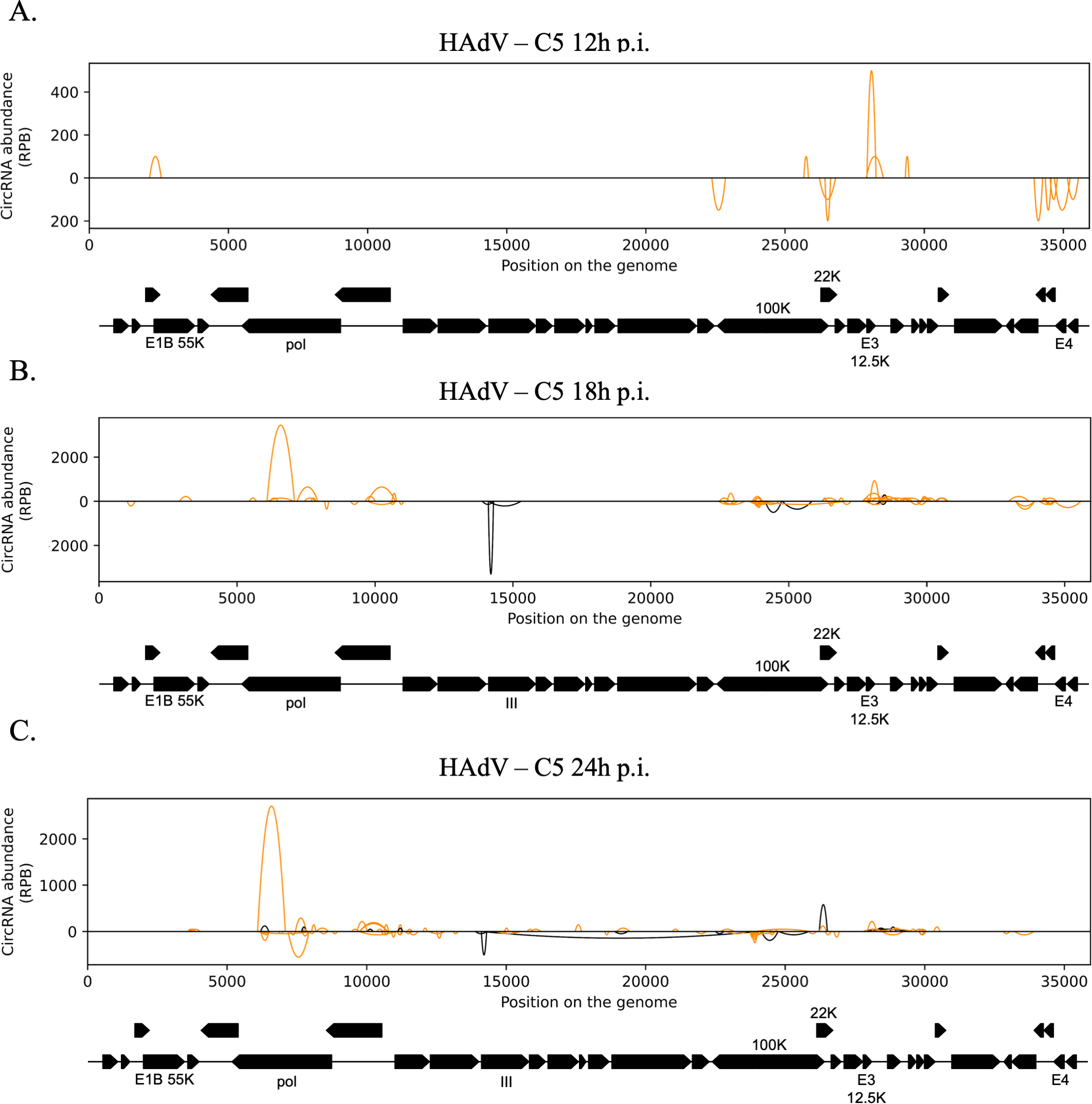

Supplement: S4 Fig — The circRNAs were mapped to the hAdV-C5 genome after 12h (A), 18h (B) or 24h (C) of infection. The infection was performed using A549 cells. Relevant ORFs were indicated under the graph on the same scale as the viral genome. The Y axis indicates the abundance of unique circRNAs in reads mapping the backsplice junctions per billion of reads mapping on the viral HAdV-C5 genome. (TIF) [file ppat.1013448.s004.tif]

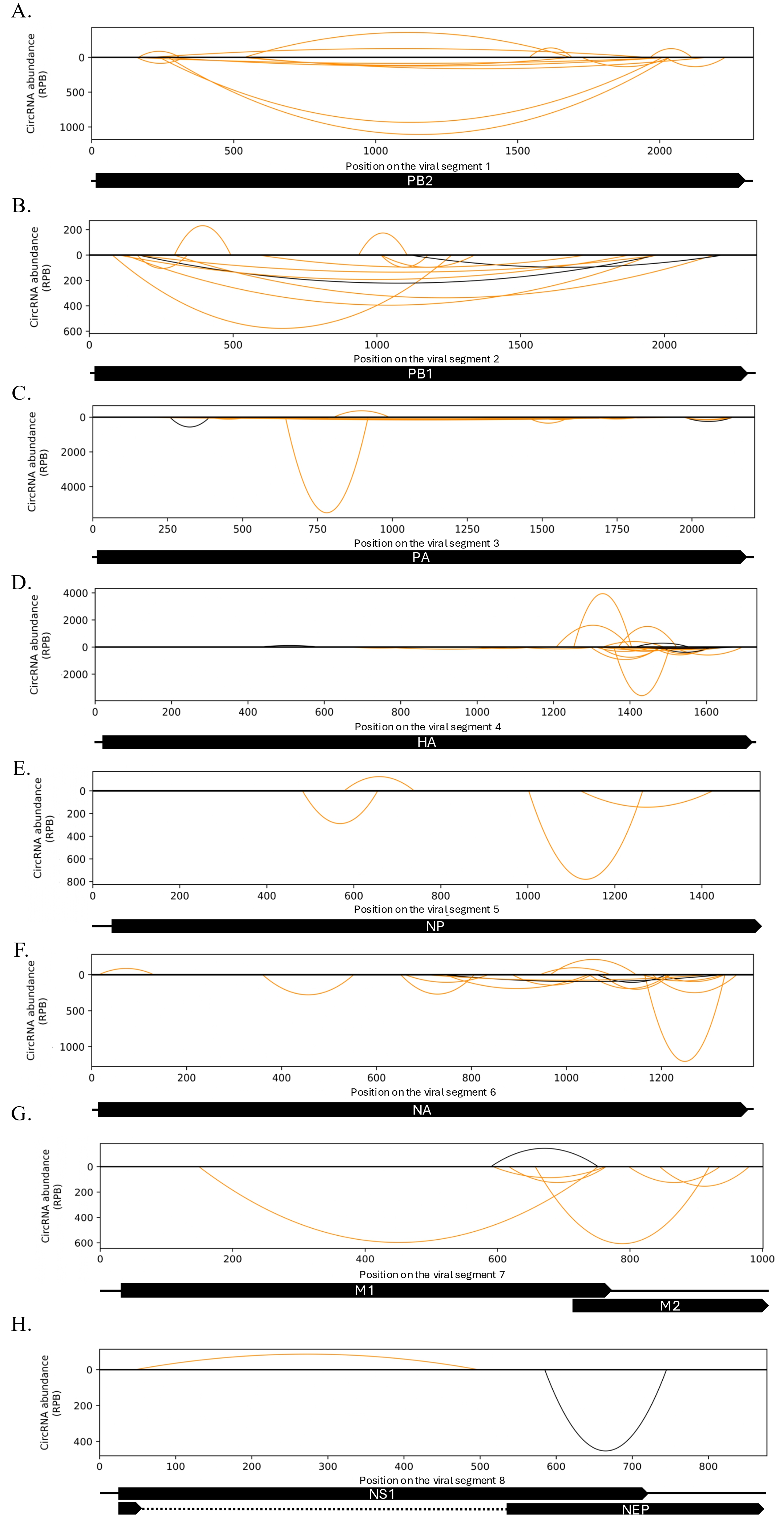

Supplement: S5 Fig — (A-H) A549 cells were infected with the H1N1 strain of IAV in the study of Min et al., 2023. The eight segments of the virus were processed through vCircTrappist and depicted in the Figure. The Y axis indicates the abundance of unique circRNAs in reads mapping the backsplice junctions per billion of reads mapping on the viral IAV H1N1 genome. (TIF) [file ppat.1013448.s005.tif]

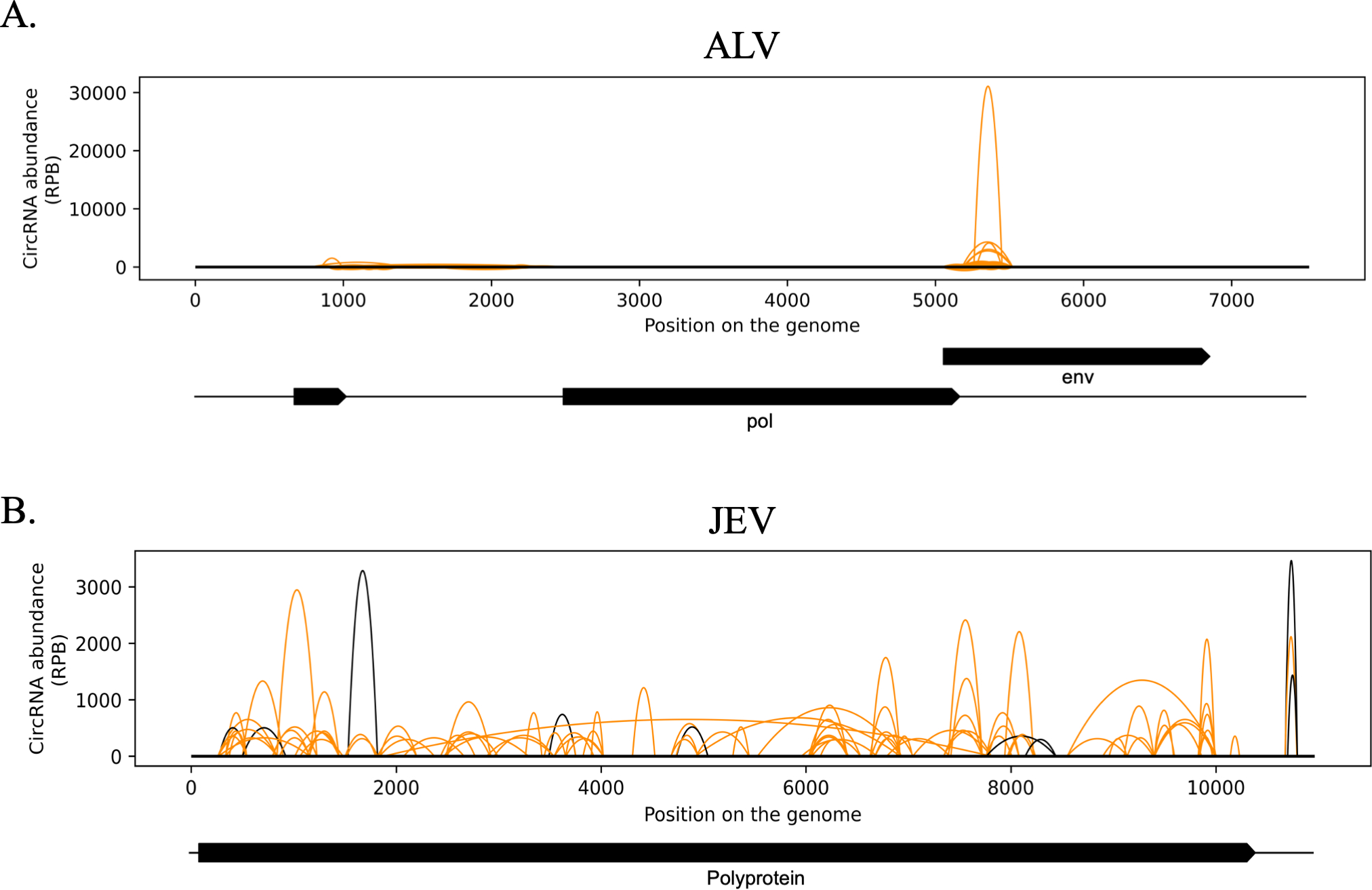

Supplement: S6 Fig — (A) Viral circRNAs identification from an ALV infection. The dataset was obtained after the infection of Chicken Embryonic Fibroblasts (CEF) in the study of Yang et al., 2022 [34]. (B) Viral circRNAs identification from a JEV infection. Mouse brains were injected with the virus and harvested after 5 days of infection in the study of Li et al, 2020 [35]. The Y axis indicates the abundance of unique circRNAs in reads mapping the backsplice junctions per billion of reads mapping on the viral genome. (TIF) [file ppat.1013448.s006.tif]

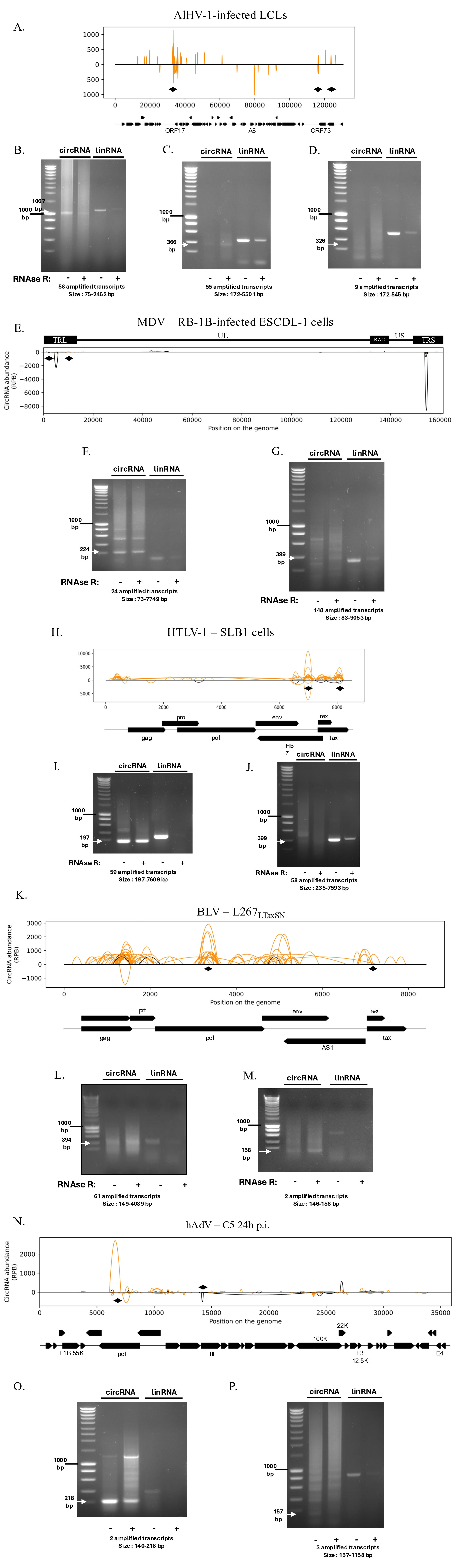

Supplement: S7 Fig — (A) Viral circRNAs expressed from an in vivo infection with AlHV-1. A lymphoblastoid cell line (LCL) from in vivo-infected calves was recovered and grown in culture. (B-C-D) RT-PCR confirmations of the loci of circRNA expression in the AlHV-1 model. Untreated samples and corresponding linear RNAs were used as controls for the RNase R treatment. White arrows were placed at the expected band size to facilitate reading. (E) Viral circRNAs expressed from an in vitro infection with MDV. The ESCDL-1 cell line was infected with the RB-1B strain of the virus and the RNAs were recovered after 6 days of infection. (F-G) RT-PCR confirmations of the loci of circRNA expression in the MDV model. Untreated samples and corresponding linear RNAs were used as controls for the RNase R treatment. White arrows were placed at the expected band size to facilitate reading. (H) Viral circRNAs expressed from an in vitro culture of productively HTLV-1-infected cells (SLB1 cell line). (I-J) RT-PCR confirmations of the loci of circRNA expression in the HTLV-1 model. Untreated samples and corresponding linear RNAs were used as controls for the RNase R treatment. The second sample represents a false positive result. White arrows were placed at the expected band size to facilitate reading. (K) Viral circRNAs expressed from BLV productively-infected cell line L267LTaxSN. (L-M) RT-PCR confirmations of the loci of circRNA expression in the BLV model. Untreated samples and corresponding linear RNAs were used as controls for the RNase R treatment. White arrows were placed at the expected band size to facilitate reading. (N) Viral circRNA identification after 24h of an infection with the HAdV-C5. The infection was performed using A549 cells. (O-P) RT-PCR confirmations of the loci of circRNA expression in the HAdV-C5 model. Untreated samples and corresponding linear RNAs were used as controls for the RNase R treatment. White arrows were placed at the expected band size to facilitate reading. (A-E-H-N) Re [file ppat.1013448.s007.tif]
